# Enhancing Shape Recovery and Mechanical Properties of Bisphenol-A-Epoxy-Based Shape Memory Polymer Composites (SMPCs) Using Amine Curing Agent Blends

**DOI:** 10.3390/polym18030373

**Published:** 2026-01-30

**Authors:** Garam Do, Sungwoong Choi, Seongeun Jang, Duyoung Choi

**Affiliations:** 1 Carbon & Light Material Application Research Group, Korea Institute of Industrial Technology (KITECH), Jeonju 54853, Republic of Korea; 2 RAMP Convergence Research Center, Korea Institute of Science and Technology (KIST), Wanju 55324, Republic of Korea

**Keywords:** shape memory polymer, amine curing agent, mechanical properties, carbon fillers, thermal stability

## Abstract

Shape memory polymer (SMP) has broad applications in various industries, including automotive, aerospace, and medical, as it can maintain a given shape and return to its original form upon exposure to external stimuli such as heat, magnetic fields, or light. However, the intrinsic limitation of epoxy results in the low thermal conductivity of SMP, which reduces the difference in temperature (ΔT) between the glass transition temperature (T_g_) and the actuation temperature, thereby negatively affecting the performance of shape recovery. In this study, the thermal stability and curing characteristics of SMP fabricated by blending Bisphenol-A epoxy with two types of amine curing agents were analyzed by thermogravimetric analysis (TGA) and differential scanning calorimetry (DSC) to establish optimal fabrication conditions. Subsequently, carbon-based fillers, graphite and 60 μm long carbon fibers, were added to fabricate shape memory polymer composites (SMPCs). The curing and mechanical properties of the SMPCs were subsequently evaluated, and the shape recovery characteristics were found to be optimal at a filler content of 3 wt%. The recovery time for the SMPC with graphite was 25 s, representing a 68.75% improvement in shape recovery time from the SMP. Furthermore, the addition of carbon fibers, with improved dispersion, led to the highest increases in tensile strength and impact strength of 24.71% and 59.36%, respectively.

## 1. Introduction

Shape memory polymers (SMPs) with the shape memory effect (SME) property to return to their original shape from a fixed temporary shape in response to a specific external stimulus such as heat [1,2], light [3,4], and magnetic fields [5] have advantages such as low cost, low density, and low weight [6,7]. With these attributes, they can be used in various industries, including automotive, aerospace, and medical fields [8,9]. Recent advances in thermally activated SMP emphasize high glass transition temperature (T_g_) polymer networks and carbon-based fillers that enhance thermal conductivity and accelerate shape recovery [10,11]. Efficient thermal conductive networks, together with 4D printing and multi-scale modeling, have significantly expanded structural design capabilities and improved predictive accuracy [12,13]. Overall, current state-of-the-art SMP research focuses on high-performance networks, carbon composite systems, and advanced fabrication approaches for next-generation aerospace, biomedical, and soft-robotic applications.

Particularly for thermally activated SMPs driven by external heat, their application in industrial sectors such as automotive and aerospace is easier compared to photo- or electro-activated SMP. Therefore, this study selected a Bisphenol-A epoxy-based thermally activated SMP as the research model.

The curing characteristics, shape recovery characteristics, and mechanical properties of SMP can be controlled by adjusting the composition of the epoxy resin and curing agent used in its fabrication, enabling the development of SMP with optimized properties for a broad array of industrial applications [14,15]. The effect of the curing agent type on SMP can be analyzed by the evaluation of curing characteristics. The T_g_, crosslink density, and degree of curing (α) are representative curing characteristics used to predict the SMP properties. The crosslink density of SMP specimens significantly influences the elastic deformation energy controlling the shape recovery characteristics [16]. In addition, T_g_ defines the operating temperature and the ΔT between the operating and ambient temperatures [17]. Moreover, by measuring α as a function of curing time, the optimal curing time for SMP fabrication can be determined.

Shape recovery time is a key characteristic of the shape recovery behavior of SMP, and it becomes faster as heat is transferred more quickly within the specimen. This heat transfer is more efficient when the thermal conductivity and ΔT values are higher. However, the epoxy resin used to fabricate SMP has a low thermal conductivity of less than 0.3 W/mK due to organic limitations [18]. This low thermal conductivity reduces the shape recovery ability of SMP and hinders its application in industries that require high thermal conductivity. Research on SMP with improved thermal conductivity is essential for a wider range of industrial applications, and the application of fillers with excellent thermal conductivity is becoming commonplace. Carbon-based fillers have been applied in many SMP studies due to their advantages such as excellent thermal conductivity and light weight [19,20]. The incorporation of high thermal conductivity fillers can improve the intrinsically low thermal conductivity of epoxy matrices, thereby accelerating shape recovery time. Graphite and carbon fiber are among the most widely employed carbon-based fillers. In the studies by L. G. Hou [21] and Jonas Ihle [22], the thermal conductivities of mild carbon fiber and graphite were measured at 129.4 and 140 W/mK, respectively; the extent to which filler addition enhances the thermal conductivity of the epoxy resin will be detailed below. In shape memory polymer composites (SMPCs) fabricated by applying these advantages, the multidimensional structure formed by the filler increases the density within the SMPC, allowing for the formation of more heat transfer channels [23]. Shape recovery performance is affected by the thermal conductivity of the SMPC, which is enhanced by the formation of heat transfer channels.

In this study, we present several distinct advancements over previous works [24,25], which primarily focused on enhancing the thermal conductivity and shape recovery performance of SMP using single amine curing systems and carbon-based fillers. We first introduce a hybrid amine curing system composed of 4,4′-Diaminodiphenylmethane, (DDM) and Jeffamine. As the contents of DDM and Jeffamine increase, the T_g_ and mechanical properties exhibit different trends. DDM functions as a hard segment that stores the elastic restoring force of the SMP, whereas Jeffamine serves as a soft segment that becomes plasticized above T_g_ and enables temporary shape fixation [26]. To determine the optimal curing agent ratio for improving SMP performance, we evaluated the curing behavior and shape system. Through this approach, we established conditions that allow systematic control of the T_g_, crosslink density, and α of the SMP, capabilities unattainable in previous studies based solely on single curing agent compositions.

Furthermore, in this study, we propose a two-step design strategy. In Step 1, we optimized the intrinsic shape recovery properties of the SMP by employing a controlled curing composition before filler incorporation, thereby elucidating mechanisms of performance enhancement that cannot be achieved through filler addition alone. In Step 2, we fabricated SMPC using carbon-based fillers, specifically graphite and recycled carbon fibers with a length of 60 μm (CF 60 μm), to further enhance the properties of the optimized SMP. The influence of fillers on the T_g_, a key factor governing shape recovery, was evaluated using differential scanning calorimetry (DSC). The thermal stability as a function of curing agent type was assessed via thermogravimetric analysis (TGA), and improvements in thermal conductivity were also analyzed. The shape recovery characteristics of the SMP were evaluated in terms of shape fixity, recovery ratio, and recovery time, while mechanical improvements at two different filler loadings were examined through tensile and impact tests. Under the optimized conditions, the resulting SMPC exhibited simultaneous improvements in shape recovery performance and mechanical properties. Collectively, these findings demonstrate a new design pathway for high-performance epoxy-based SMP and SMPC systems by integrating curing agent composition design with filler-based reinforcement.

## 2. Materials and Methods

### 2.1. Materials

**Figure 1:**
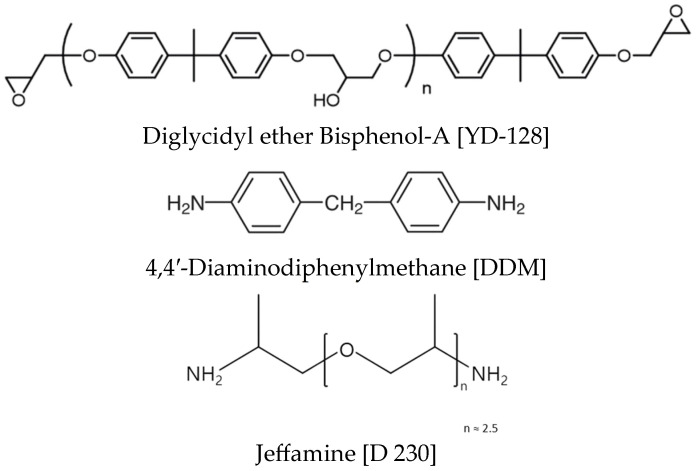
Chemical structure of epoxy resin and curing agents.

Bisphenol-A based epoxy resin, YD-128, was used for the fabrication of SMPC and was purchased from Kukdo Chemical in South Korea. DDM with an aromatic structure and Jeffamine with an aliphatic structure were used as curing agents, which were purchased from TCI and Huntsman (USA). The densities of the epoxy resin and the two curing agents were 1.16 g/cm^3^, 1.08 g/cm^3^, and 0.95 g/cm^3^, respectively, and the chemical structures of these materials are shown in Figure 1. Additionally, carbon-based fillers were used to improve thermal conductivity. Graphite with a particle size range of 100–300 μm was purchased from Sigma Aldrich (USA), and CF 60 μm was purchased from Fiberman (South Korea). The mixing ratios of the epoxy resin and the two curing agents used in the fabrication of SMP are shown in Table 1. The mixing ratios of the curing agents combined with the epoxy resin, used to fabricate the SMP, are shown in Table 2. Within a total curing agent content of 30 wt%, the mixing ratio of DDM and Jeffamine was varied, and the resulting T_g_, shape recovery characteristics, and mechanical properties were evaluated.

**Figure 2:**
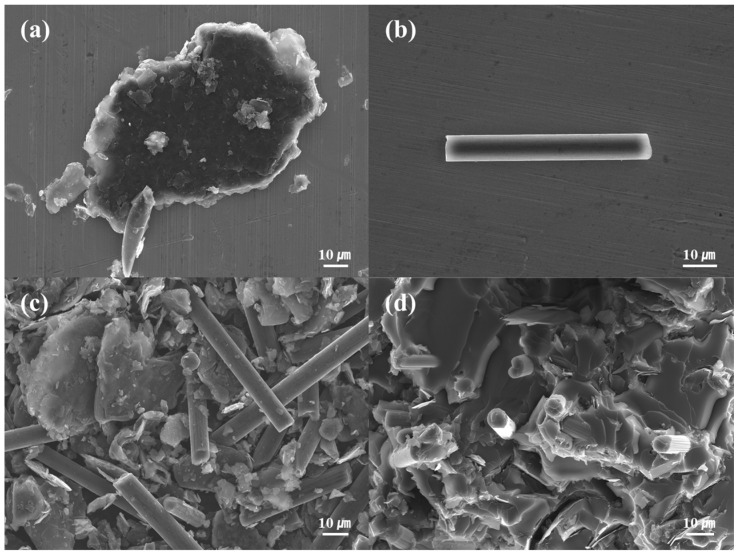
FE-SEM images of the fillers. (**a**) Graphite. (**b**) CF 60 μm. (**c**) Hybrid. (**d**) Fracture surface of 20 wt% hybrid SMPC.

FE-SEM images of carbon-based fillers applied to the SMP are shown in Figure 2. Graphite with a diameter of 100–300 μm is shown in Figure 2a. In Figure 2b, CF 60 μm can be observed. The fracture surface of SMPC with 20 wt% hybrid filler is shown in Figure 2d. The carbon fibers dispersed within the epoxy matrix are uniformly distributed. Additionally, the fractured fiber surfaces indicate strong interfacial adhesion with the epoxy matrix, facilitating effective thermal conductivity transfer and mechanical load distribution.

To prepare the solution for SMPC fabrication, YD-128 was preheated at 110 °C, and then the curing agents and fillers were added. The dispersion and degassing of the filler and solution were performed using a planetary mixer (ARE-310) from Thinky, Japan, at 2000 and 2200 rpm for several minutes. The specimens used for measurements were fabricated by casting the solution into aluminum and 3D-printed molds which were heated to the curing temperature. All measurements were conducted after allowing the specimens to stabilize for one day following fabrication.

### 2.2. Methods

#### 2.2.1. Field Emission Scanning Electron Microscopes

To confirm the shape and size of the filler added to the SMPC, images of graphite and CF 60 μm were taken using field emission scanning electron microscopes (FE-SEMs, JMS-7100F, JEOL Ltd., USA).

#### 2.2.2. Differential Scanning Calorimetry

T_g_ of SMP and SMPC were analyzed by differential scanning calorimetry (DSC 250, TA Instrument, USA). DSC measurements were performed under a N_2_ atmosphere, and 3–6 mg samples were heated from 40 to 250 °C at a ramping rate of 10 °C/min.

#### 2.2.3. Degree of Cure (α)

The degree of cure at various curing times, depending on the type of curing agent used in the fabrication of SMPC specimens, was calculated using Equation (1).

(1) $$\alpha =\frac{{∆H}_{t}}{{∆H}_{R}}\times 100\left(\%\right)$$

ΔH_t_ is the heat of reaction corresponding to a specific curing time and ΔH_R_ is the total heat of the uncured resin.

#### 2.2.4. Thermogravimetric Analysis

The thermal stability and residual mass content of SMP prepared using single and mixed curing agents were analyzed by thermogravimetric analysis (TGA, SDT 650, TA Instrument, USA). The TGA measurements were performed under a N_2_ atmosphere, and 5–8 mg samples were heated from 40 to 700 °C at a ramping rate of 10 °C/min.

#### 2.2.5. Thermal Conductivity

To evaluate the enhancement in thermal conductivity as a function of the filler type and content, thermal conductivity was measured by a thermal conductivity measurement system (TPS 2500S, Hot Disk, Sweden). The samples were polished with sandpaper and were used after completing the drying process.

#### 2.2.6. Shape Recovery Characteristics

To evaluate the shape recovery characteristics of a 100 mm × 20 mm × 1 mm sample cured at 150 °C, shape fixation, recovery ratio, and recovery time were measured. The deformed specimen was fixed using a 3D mold, where the fixed angle was defined as θ_fixed_, the maximum bending angle as θ_max_, and the recovery angle as θ_i_. Using these values, the ratios of the shape fixation and recovery were calculated with Equations (2) and (3), respectively.

(2) $$Shape\;fixation\;ratio=\frac{\theta_{max}{-\theta }_{fixed}}{\theta_{max}}\times 100\left(\%\right)$$

(3) $$Shape\;recovery\;ratio=\frac{\theta_{max}-\theta_{i}}{\theta_{max}}\times 100\left(\%\right)$$

In this study, the recovery time was evaluated based on the recovery angle perpendicular to the ground to eliminate the effect of rapid recovery induced by gravity.

#### 2.2.7. Tensile Strength Test

To evaluate the mechanical properties of the fabricated SMP and SMPC, tensile strength measurements were performed. The specimens were fabricated into a dog bone shape of 165 mm × 13 mm × 3.2 mm according to the ASTM D638 standard [27], and measurements were conducted using a universal testing machine (Fatigue Testing Machine 8801, Instron, USA) at a testing speed of 5 mm/min.

#### 2.2.8. Impact Strength Test

To analyze the mechanical properties based on the content of Jeffamine, which has flexible polymer chains, impact strength was measured using an Izod impact testing machine (Model Impact 104, Tinius Olsen, USA). The samples were fabricated according to the ASTM D256 standard [28], with an overall length of 63.5 mm, a notch width of 11.2 mm, and a thickness of 3.2 mm. The impact strength variation as a function of curing agent content was analyzed.

## 3. Results and Discussion

### 3.1. Analysis of the Cure Properties of the SMP

The controlled blending of two curing agents with distinct molecular structures alters the free volume within the SMP matrix, thereby enabling direct modulation of the crosslink density, which critically governs the shape memory behavior. Consequently, the balance between crosslink density and free volume, which exhibit an inverse relationship [29,30], determines the shape fixity and recovery kinetics of the SMP. By leveraging this relationship, SMPs with tailored thermomechanical properties can be designed, as verified through thermal and curing analyses such as TGA, DSC, and α measurements.

#### 3.1.1. Differential Scanning Calorimetry

**Figure 3:**
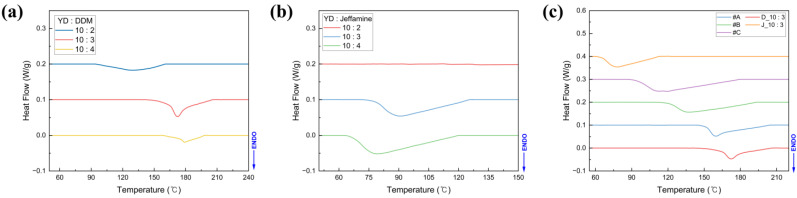
DSC results of SMP according to the different types of curing agent: (**a**) DDM, (**b**) Jeffamine, (**c**) DDM + Jeffamine.

**Figure 4:**
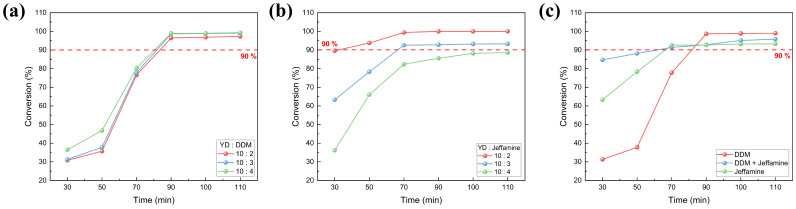
SMP’s conversion rate as a function of curing time: (**a**) single DDM, (**b**) single Jeffamine, (**c**) #C (DDM/Jeffamine = 0.5:2.5).

The T_g_ is a critical parameter in shape memory polymers, as it defines the transition between the rigid glassy state and the flexible rubbery state, thereby governing both shape fixation and shape recovery behavior [31]. Figure 3 presents the DSC results for SMP prepared with different curing agents. The T_g_ values for each DSC curve were calculated using the midpoint method with auxiliary baseline construction, and the resulting T_g_ for different curing agent mixing conditions are summarized in Table 3. When DDM was employed as the sole curing agent (Figure 3a), T_g_ increased progressively with higher DDM content. This can be attributed to the short and rigid aromatic backbone of DDM, which promotes dense crosslinking within the epoxy matrix. The higher crosslink density restricts polymer chain mobility, thereby requiring greater thermal energy for the transition into the rubbery phase. This trend has been widely reported for aromatic curing systems in epoxy resins [32]. In contrast, as shown in Figure 3b, the T_g_ significantly decreased with increasing Jeffamine content. This is because the flexible and long aliphatic polymer chains with ether linkages reduce the crosslink density during the curing process [33]. The crosslink density decreases by increasing the interchain distance and enhancing segmental mobility. Consequently, T_g_ decreases as the flexible domains dominate, facilitating shape recovery at lower activation temperatures [34,35]. In epoxy curing systems, once the network has reached a sufficiently high degree of cure, no additional reactive amine groups remain available to participate in further polymerization. Consequently, no exothermic peak associated with cure progression is observed in the DSC thermogram of a fully cured specimen [36,37,38,39,40]. In Figure 3b (YD/Jeffamine = 10:2), the sample cured for 100 min exhibits such behavior: the curing reaction is essentially complete, and therefore the DSC curve appears flat with no discernible exothermic peak. This observation is consistent with the degree-of-cure analysis presented in Figure 4.

In the case of Figure 3c with the mixed curing agent, a tunable T_g_ was obtained by varying the ratio of DDM to Jeffamine. The lowest T_g_ was observed for condition #C, with a DDM-to-Jeffamine ratio of 0.5:2.5, confirming that the flexible Jeffamine segments largely dictate the thermal transition. The ability to tailor T_g_ through hybrid curing chemistry is particularly valuable for engineering SMP with application-specific activation temperatures, such as biomedical stents (near body temperature) or aerospace deployable structures (above 100 °C).

#### 3.1.2. Degree of Cure

Due to the nature of SMP, where shape recovery is driven by the elastic recovery energy stored in polymer chains, analyzing curing characteristics such as crosslink density and degree of cure is essential. The degree of cure can predict the crosslink density over curing time. In order to determine the optimal curing conditions for SMP with superior shape recovery characteristics, the peak area obtained from DSC analysis was calculated using Equation (1). The degree of cure of specimens cured for 30 to 110 min was calculated through DSC measurements as shown in Figure 4. The results indicate that the degree of cure increases proportionally with the content of DDM, which has a short polymer chain, whereas it decreases with the content of Jeffamine, which has a long polymer chain. This is because the long polymer chains increase the distance between crosslinking points during curing, ultimately reducing the crosslink density.

In the case of Figure 4c, the tertiary amine generated during curing acted as a catalyst in the mixed curing agent system, resulting in curing at a lower temperature compared to the system with a single curing agent [41]. Therefore, under the same curing temperature, the initial curing degree up to 50 min was superior compared to other curing conditions. In all curing systems, as the curing time increased, the degree of cure increased. For the SMP fabricated with a 10:3 content ratio, it was confirmed that more than 90% of the curing occurred after 90 min of curing time. These findings demonstrate that hybrid curing not only tunes T_g_ but also accelerates cure kinetics, a feature beneficial for industrial processing.

#### 3.1.3. Thermogravimetric Analysis

**Figure 5:**
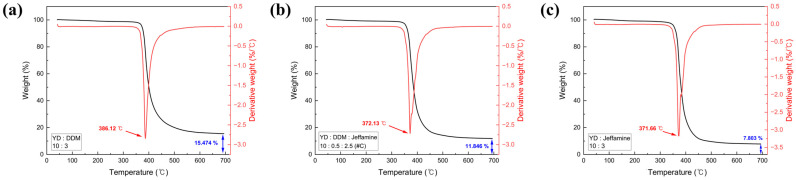
TGA curves of specimens with different curing agents: (**a**) single DDM at a 10:3 ratio, (**b**) YDJ_#C, and (**c**) single Jeffamine at a 10:3 ratio.

As shown in Figure 5, derivative thermogravimetry (DTG) peak temperature and residual mass of the SMP increased with increasing DDM content, indicating enhanced thermal stability. This behavior is attributed to the higher crosslink density achieved at high DDM content, which increases the energy required for thermal decomposition. Moreover, the aromatic curing agent DDM promotes char formation during thermal decomposition, thereby increasing the residual mass. Thus, higher DDM loadings in SMP correlate with progressively greater residual char yields [42,43,44]. Figure 3 shows that the T_g_ also increases with DDM content, consistent with the formation of a denser crosslinked network that restricts segmental mobility.

However, the improved thermal stability and corresponding increase in stiffness is expected to delay shape recovery. In highly cured SMP, the denser crosslinked network restricts polymer chain mobility during recovery, thereby prolonging recovery time. Therefore, optimization of the curing conditions is crucial to achieve both enhanced thermal stability and rapid shape recovery.

#### 3.1.4. Shape Recovery Characteristics

**Figure 6:**
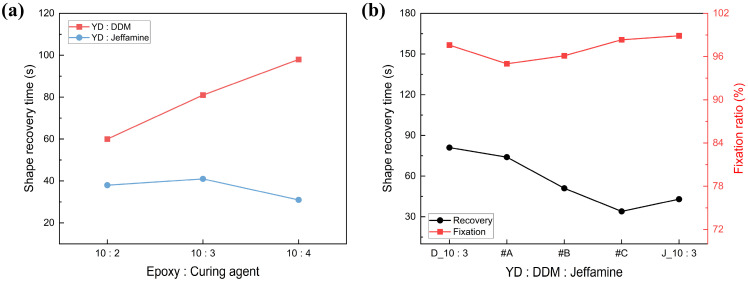
Shape recovery time of SMP with various contents of curing agent: (**a**) single curing agent, (**b**) mixed curing agent.

As previously described, the shape recovery characteristics of SMP are strongly affected by the curing degree and T_g_ of the specimen, and the curing characteristics were analyzed by DSC. Based on this, the shape recovery characteristics of SMP produced by each curing agent were evaluated and are shown in Figure 6. In the case of DDM, the shape recovery time was slowed down due to high crosslink density, which leads to small elastic recovery force stored in the polymer network due to the shortening of the polymer chain length between the crosslinking points [45,46]. On the other hand, in the case of Jeffamine, whose crosslink density decreases with increasing content, the elastic recovery force that can be stored increases, and the shape recovery time becomes faster with increasing content. The same trend was found for SMP with mixed curing agents, with SMP with more stored elasticity not only having a better shape recovery time but also a better shape fixation ratio.

As shown in Figure 6b, a balance was achieved in which the polymer exhibited both high recovery speed and good fixation capability. The tunability of recovery kinetics through curing agent ratios aligns with recent studies emphasizing the importance of molecular design in SMP performance optimization [47].

### 3.2. Evaluation of Mechanical Properties of SMP

#### 3.2.1. Tensile Strength Test of SMP

**Figure 7:**
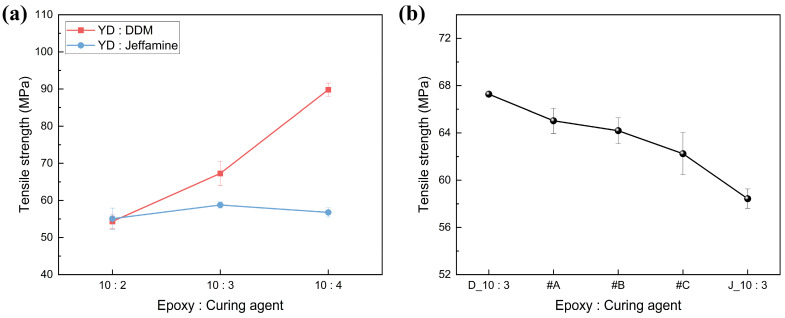
Tensile strength of SMP with various contents of curing agent: (**a**) single curing agent, (**b**) mixed curing agent.

The mechanical properties of SMP are significantly influenced by the structure of the curing agent, and the fabrication of SMP with the desired properties is achieved by selecting the appropriate curing agent type. In this study, the effects of DDM and Jeffamine on the mechanical properties of SMP were investigated through the measurement of the tensile strength and impact strength of SMP cured with each curing agent individually, as well as in combination. The effect of crosslink density, which varies due to the structural differences in curing agents, on the tensile strength trend is shown in Figure 7. In the case of DDM, where the crosslink density increases with the curing agent content, it was observed that the tensile strength increased as the curing agent content increased. This is because the high crosslink density formed within the specimen effectively contributed to the distribution of tensile stress. On the other hand, in the case of Jeffamine, where the crosslink density and curing degree decrease as the content increases due to the long polymer chains, the tensile strength decreased as the curing agent content increased. The addition of soft materials with good flexibility, such as Jeffamine, reduces the tensile strength of epoxy resins [48,49]. Therefore, when the two curing agents were blended, the tensile strength decreased with increasing content of Jeffamine, which has good flexibility in long chains.

However, for the SMP with mixed curing agents, the tensile strength did not decrease proportionally with the added Jeffamine. Specifically, for #B and #C, the tensile strength was higher than expected. This can be attributed to the incorporation of DDM, which has excellent crosslinking density and helped mitigate the strength reduction.

#### 3.2.2. Impact Strength Test of SMP

**Figure 8:**
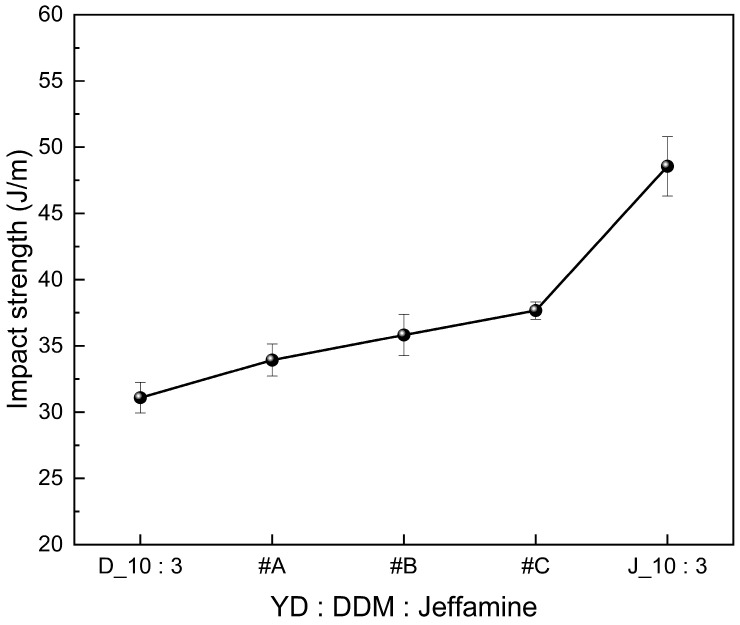
Impact strength of SMP with various amounts of mixed curing agent.

Jeffamine, which forms long-length linkages, contains ether groups in its structure that contribute to improving the impact strength. Furthermore, the addition of Jeffamine increases the flexibility of epoxy resin, and it effectively aids in stress distribution and relaxation when impact loads are applied [50,51]. So, in Figure 8, the impact strength of SMP was increased by Jeffamine content in mixed amine agents.

### 3.3. Analysis of the Thermal Characteristics of the SMPC

#### 3.3.1. Thermal Conductivity

Thermal conductivity influences recovery kinetics in SMP by determining how efficiently heat is distributed throughout the material. In this study, SMPCs were fabricated by incorporating graphite, a carbon-based filler with excellent thermal conductivity, and 60 µm long carbon fibers. The content ratios of the added fillers are shown in Table 4.

**Figure 9:**
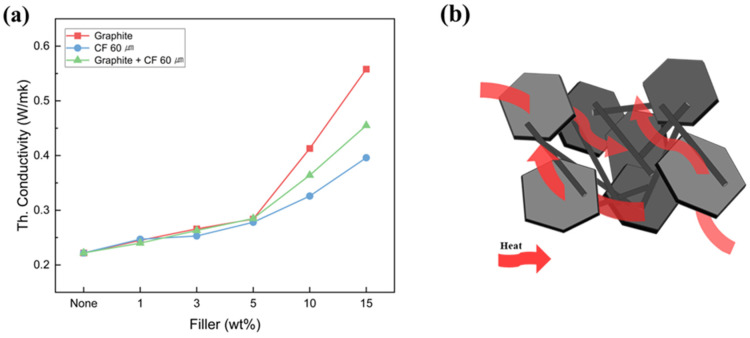
(**a**) A thermal conductivity graph as a function of the content of single and mixed fillers and (**b**) schematic of heat transfer pathways via fillers.

The thermal conductivity of the SMPC as a function of filler type and content is shown in Figure 9a. The thermal conductivity increased in the order of CF 60 µm < hybrid < graphite, and the addition of fillers enhanced the thermal conductivity of the SMP from 0.222 W/mK to a maximum of 0.558 W/mK, corresponding to a 151.35% increase. As shown in Figure 9b, at concentrations exceeding 5 wt%, a dense filler network was formed within the polymer matrix, creating efficient pathways for heat transfer and significantly enhancing thermal conductivity.

As reported in numerous studies [20,30,52], the incorporation of carbon-based fillers directly influences the T_g_ of SMPs. The fillers create physical constraint points and interfacial regions within the matrix, where the packing density and local free volume of polymer chains are rearranged in ways that differ from those of the native network. These localized variations in free volume modify the segmental mobility and relaxation dynamics of the polymer chains, ultimately leading to a shift in T_g_ [53]. In particular, carbon-based fillers can exhibit a dual effect on chain mobility near the interface due to their high surface energy and rigid structural characteristics. Depending on the filler morphology, surface chemistry, dispersion state, and the initial crosslink density of the polymer network, they may either enhance or suppress molecular mobility in the interfacial region [53,54,55,56]. In this study, we also observed behavior consistent with these previous reports, and our DSC analysis experimentally confirmed that the increase in local chain mobility at the filler–matrix interface contributes to the reduction in T_g_. This improved thermal conductivity reduces the energy required for the composite to transition from the glassy to the rubbery state, thereby contributing to a lower T_g_ in the SMPC [57].

#### 3.3.2. T_g_ Modification by Fillers

**Figure 10:**
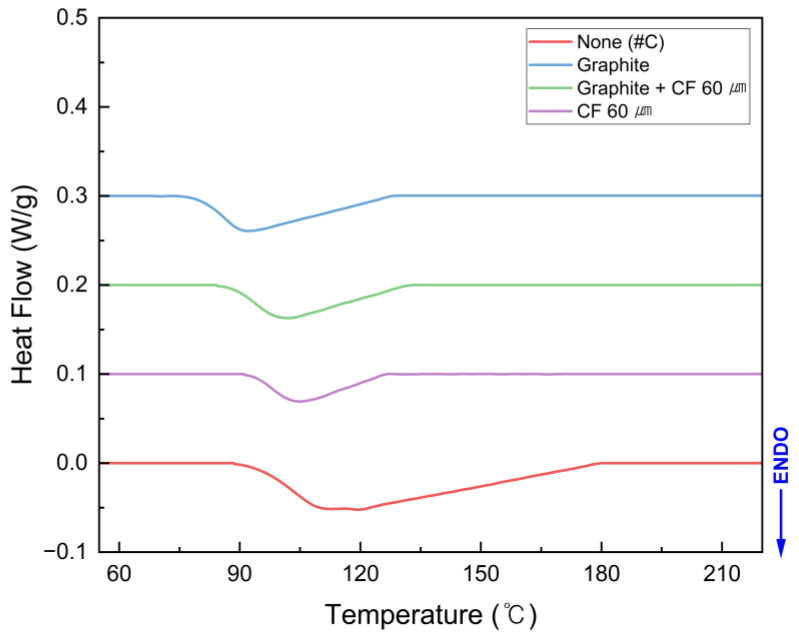
DSC results of the SMP and SMPC according to the single and mixed filler.

The SMPC with 3 wt% filler content was used for the analysis of the effect of filler on the shape recovery characteristics, and the DSC graphs of different filler types are shown in Figure 10. It shows the DSC curve showing the change in T_g_ with filler application. The T_g_ of the non-filler SMPC was 102.33 °C, while that of the graphite-filled SMPC was 85.51 °C, resulting in a maximum reduction of 16.82 °C. The decrease in T_g_ occurred in the order of fillers with higher thermal conductivity. This is because the superior thermal conductivity of the filler facilitated heat transfer and localized chain mobility around filler–matrix interfaces. Therefore, the T_g_ of the SMPC shifted to a lower value with the addition of fillers [52,58]. This lower T_g_ increases the temperature difference (ΔT) between the operating temperature and the transition temperature.

A high ΔT enhances the shape recovery rate of SMPC. Based on the above results, the graphite-filled SMPC in condition #C, which exhibits the lowest T_g_, is expected to demonstrate the fastest shape recovery time.

### 3.4. Shape Recovery Characteristics of SMPC

**Figure 11:**
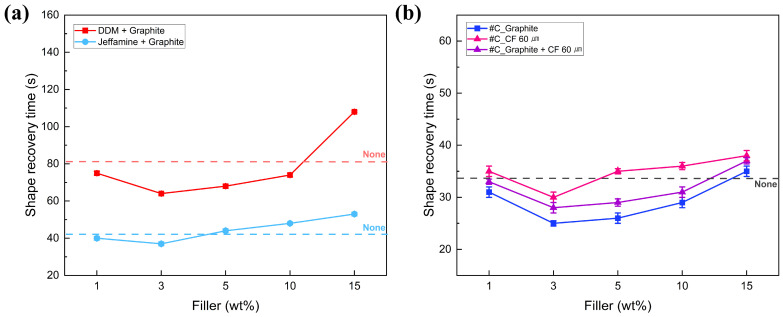
Shape recovery time of SMP and SMPC according to various types of curing agents and fillers. (**a**) Single curing agent. (**b**) Mixed curing agent in #C condition.

To analyze the effect of filler application on the shape recovery characteristics of SMPC, shape recovery tests were conducted at a temperature of T_g_ + β °C, and the results are summarized in Figure 11. In the case of single curing agent SMPC shown in Figure 11a, the shape recovery time of graphite-filled SMPC was higher in Jeffamine-based samples than in DDM-based ones, showing a 25.93% improvement compared to non-filler DDM SMP. For Jeffamine-based SMP, the application of graphite enhanced the shape recovery time by up to 17.02%. These results confirm that the addition of fillers effectively enhances the shape recovery time of SMP.

The addition of filler to the SMPC improved the shape recovery time compared to the SMP, which may be caused by the enhanced thermal conductivity of the filler. However, as the filler content increased, the recovery time also progressively lengthened. This delay is due to the van der Waals forces between the fillers and agglomeration, which dissipate the stored elastic recovery energy of the specimen, thereby suppressing the shape recovery rate. Under all conditions, the fastest shape recovery time was observed at a 3 wt% filler content. As observed in Figure 9a, thermally conductive filler-incorporated SMPC exhibited faster a shape recovery mechanism. This can be attributed to the reduction in T_g_ caused by filler addition, which increased the ΔT value, thereby enhancing recovery speed. Specifically, #C_3 wt% graphite showed a maximum improvement of 26.47% compared to the non-filled sample. Moreover, #C with 3 wt% graphite filler samples had a 69.14% faster recovery time than the non-filled DDM SMP, which exhibited the slowest recovery time, as shown in Figure 6 and Figure 11a.

**Figure 12:**
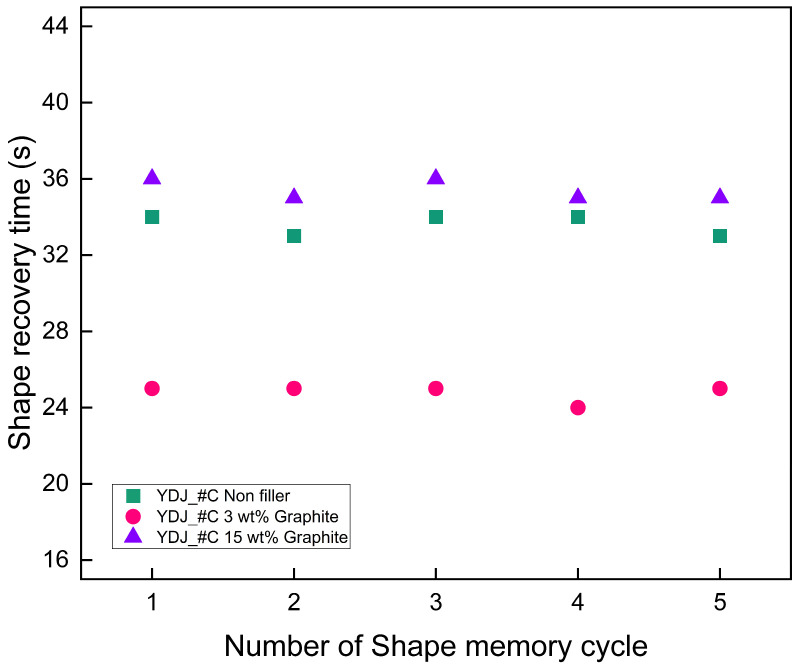
Shape recovery cycle test of 0, 3, and 15 wt% graphite SMPC.

Figure 12 shows the results of shape recovery cycle tests conducted to evaluate the durability of SMPC containing 0, 3, and 15 wt% graphite. Each specimen underwent five successive thermal cycles, each comprising isothermal heating at 170 °C for 2 min followed by cooling at room temperature (25 °C) for 5 min. Across all cycles, the recovery time of every specimen varied by no more than ±1 s, indicating negligible performance degradation and demonstrating that the SMPC can sustain repeated actuation reliably.

**Figure 13:**
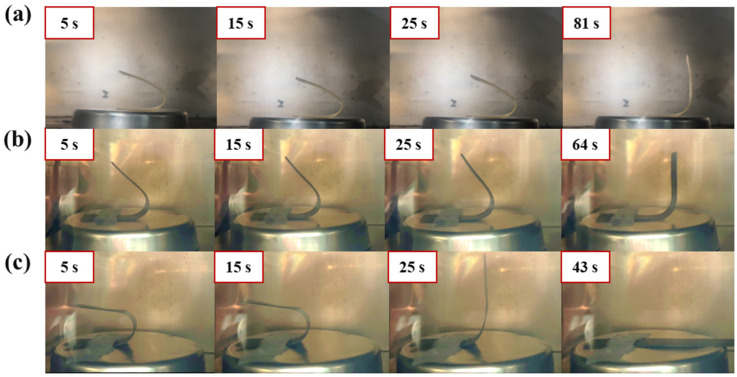
The snapshots of the shape recovery performance of SMP and SMPC. (**a**) Single curing agent (YD:DDM = 10:3) SMP. (**b**) Single curing agent DDM SMPC with 3 wt% graphite. (**c**) Mixed curing agent #C (DDM/Jeffamine = 0.5:2.5) SMPC with 3 wt% graphite.

Snapshots of the shape recovery behavior of SMP and SMPC are shown in Figure 13. The snapshots sequentially depict the shape recovery behavior of DDM_SMP, DDM_graphite 3 wt%, and #C with graphite 3 wt% SMPC. The recovery time was measured based on the moment when the recovering specimen became perpendicular to the ground. Snapshots were taken at 5, 15, and 25 s, as well as at the moment when the specimen fully recovered to a perpendicular position. The shape recovery behavior of the SMP and SMPC was different depending on the fabrication conditions, and it was possible to control the shape recovery characteristics by controlling the fabrication conditions of SMPC through curing agents and fillers.

### 3.5. Evaluation of Mechanical Properties of SMPC

#### 3.5.1. Tensile Strength Test of SMPC

**Figure 14:**
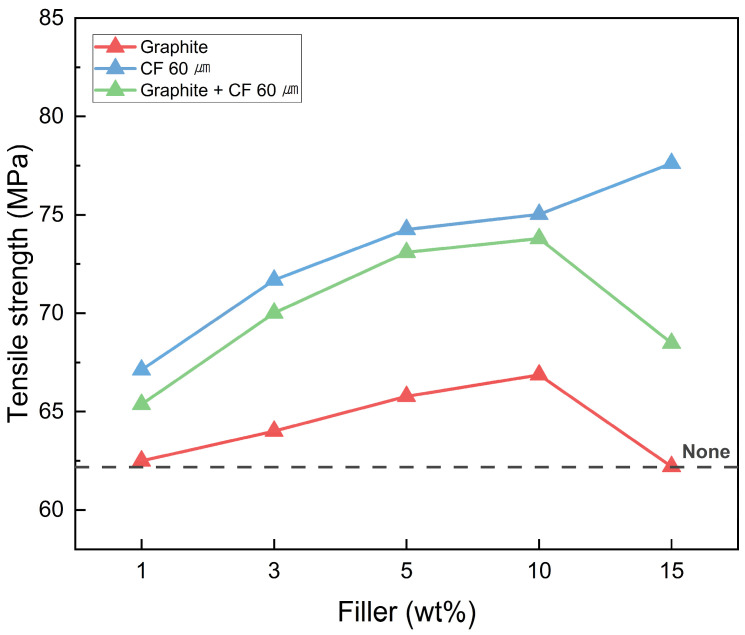
Tensile strength of SMPC with various amounts of filler.

The carbon-based fillers used in this study not only enhanced shape recovery characteristics through their excellent thermal conductivity but also contributed to improving mechanical properties, such as tensile strength [59]. The incorporation led to the formation of 2D and 3D reinforced networks within the epoxy matrix, which effectively redistributed the applied tensile stress. As the filler content increased, these networks became more continuous and robust, thereby enhancing load transfer efficiency and suppressing localized stress concentrations. Consequently, the tensile strength of SMP fabricated under #C curing conditions was evaluated as a function of filler type and content, as shown in Figure 14.

The tensile strength improved in the order of CF 60 μm > hybrid > graphite, with a maximum enhancement of 24.71% compared to the non-filled sample. In the case of the hybrid filler, the mechanical properties were enhanced to a level similar to that of CF 60 μm, which exhibited the highest improvement, despite graphite alone showing relatively lower enhancement. This result confirms that the combined application of fillers is more effective in achieving better mechanical properties than the use of graphite alone.

#### 3.5.2. Impact Strength Test of SMPC

**Figure 15:**
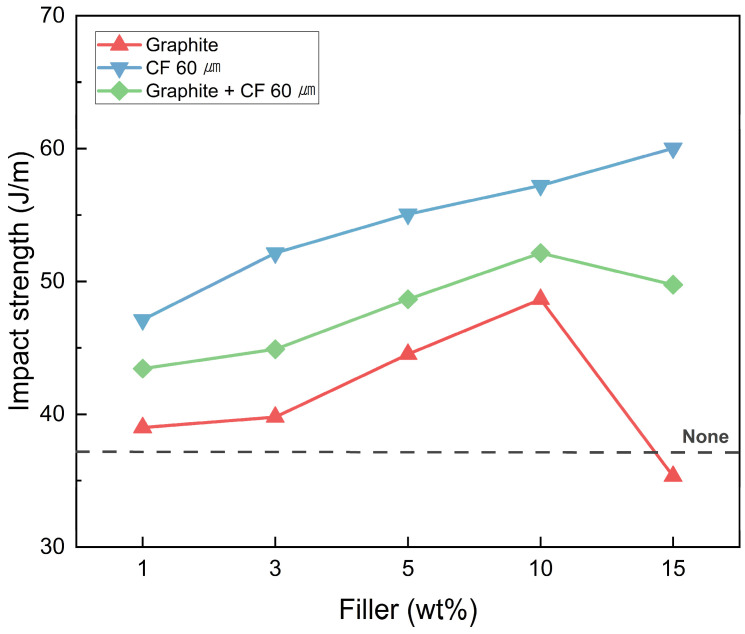
Impact strength of SMPC with various amounts of filler.

The graph measuring the impact strength of SMPC is shown in Figure 15. The application of fillers improved the impact strength of SMPC, with carbon fibers being the most effective. This can be attributed to the increase in the crack propagation length required to cause failure in the composite material due to the application of carbon fiber [60,61]. Similarly to tensile strength, the multidimensional structure formed within the SMPC specimen by the filler effectively contributes to the dispersion of impact loads applied to the specimen. The impact strength was observed in the order of CF μm > hybrid > graphite, with a maximum improvement of 59.36% compared to none. However, at high filler contents, this effect becomes relatively insignificant for the same reason as in tensile strength. This confirms that the appropriate application of filler effectively enhances the impact strength of SMPC and the mechanical properties of SMPC can be controlled by adjusting the filler content.

**Figure 16:**
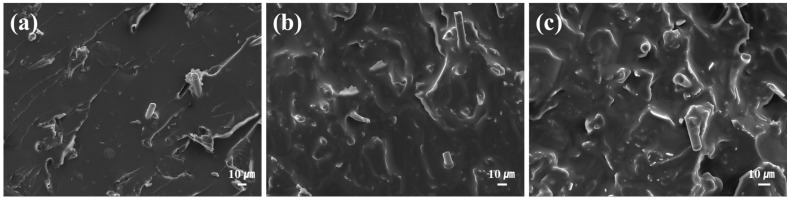
SEM images of fracture surfaces of #C (DDM/Jeffamine = 0.5:2.5) SMP according to hybrid filler content: (**a**) 3 wt %, (**b**) 5 wt%, and (**c**) 10 wt%.

Figure 16 shows the fracture surfaces of impact strength specimens with varying contents of hybrid filler. As the filler content increases, more graphite and CF are observed to be dispersed on the surface. Adding the appropriate content of filler and improving dispersion is essential for improving the mechanical properties of SMPC. Compared to graphite particles (100–300 μm), CFs with a length of 60 μm exhibit better dispersion due to their smaller size. It has been reported that both smaller graphite particles and shorter CFs exhibit better dispersion characteristics in polymer matrices [62,63]. Therefore, in the evaluation of tensile and impact strength, SMPC reinforced with CF exhibited better mechanical properties than those with graphite.

**Figure 17:**
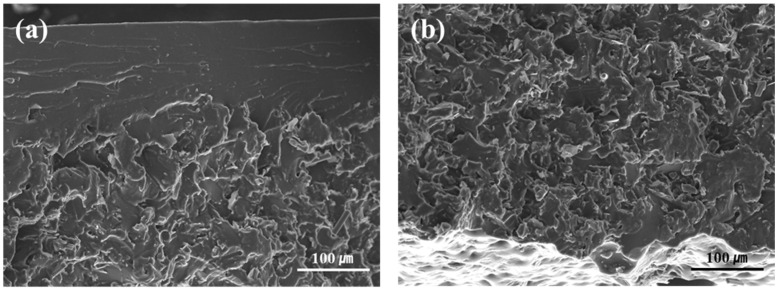
SEM images of the top region (**a**) and bottom region (**b**) of the #C (DDM/Jeffamine = 0.5:2.5) SMP containing 15 wt% hybrid filler.

Figure 17 presents the fracture surface of the impact test specimen containing 15 wt% hybrid filler in the #C condition. A clear variation in filler distribution was observed throughout the specimen’s cross-section, characterized by filler-deficient regions in the top region and filler agglomeration in the bottom region. This heterogeneity in filler dispersion is attributed to the gravitational sedimentation of filler particles onto the specimen’s bottom surface during the curing process. Such agglomeration behavior, commonly observed at high filler loadings, induces localized stress concentrations under applied load and disrupts the continuity and homogeneity of the polymer matrix [64]. Consequently, the efficiency of stress transfer between the matrix and the filler is significantly diminished, serving as a critical factor contributing to the degradation of mechanical properties in high-filler-content SMP composites.

## 4. Conclusions

In this study, SMPCs were fabricated by mixing amine-based curing agents with different structures and applying carbon-based fillers to enhance the organic limitations of SMP, including low thermal conductivity and mechanical properties. To establish the optimal mixing ratio of curing agents for fabricating SMPC, the curing characteristics, shape recovery characteristics, and mechanical properties were evaluated as a function of the content of the two curing agents. The thermal properties, shape recovery characteristics, and mechanical properties of SMPC fabricated under the established optimum conditions were evaluated through DSC, TGA, tensile strength, and impact strength, and the shape recovery rate could be improved by up to 69.14%, tensile strength by 24.71%, and impact strength by 59.36%. By controlling the mixing ratio of each curing agent and filler, the T_g_ of the SMPC could be adjusted to the desired temperature range. It was also confirmed that the T_g_ could be precisely controlled to the desired value, while simultaneously improving the shape recovery characteristics and mechanical properties.

## Figures and Tables

**Table 1:** The mixing ratio of epoxy resin and curing agents.

Epoxy	Curing Agents	
YD-128	DDM	Jeffamine
10	2	
	3	
	4	

**Table 2:** The mixing ratio of epoxy resin and curing agents.

Epoxy	Curing Agents		Total	Name
YD-128	DDM	Jeffamine		
	3	0	10:3	D_10:3
	2.5	0.5		#A
10	1.5	1.5		#B
	0.5	2.5		#C
	0	3		J_10:3

**Table 3:** Glass transition temperature of SMP as a function of curing agent composition.

T_g_ (°C)					
YD/DDM		YD/Jeffamine		YD/DDM/Jeffamine	
10:2	104.67	10:2	-	#A	155.33
10:3	166.44	10:3	82.79	#B	127.98
10:4	176.23	10:4	71.23	#C	102.74

**Table 4:** The content ratio of each single and mixed filler.

	Filler Type	Total Content (wt%)
Single	Graphite	1, 3, 5, 10, 15
	CF 60 µm	
Hybrid	Graphite + CF 60 µm	1, 3, 5, 10, 15
	(1:1)	

## Data Availability

Data are contained within the article.
